# Seasonal Effects on Pathogenicity and Biocontrol Management of *Botryosphaeria* Dieback in *Vitis vinifera* L. cv. Cabernet Sauvignon and Sauvignon Blanc Under Field Conditions

**DOI:** 10.3390/plants15050728

**Published:** 2026-02-27

**Authors:** Diyanira Castillo-Novales, Alejandra Larach, Paulina Vega-Celedón, Michael Seeger, Ximena Besoain

**Affiliations:** 1 Laboratorio de Fitopatología, Escuela de Agronomía, Pontificia Universidad Católica de Valparaíso, San Francisco s/n La Palma, Quillota 2260000, Chile; alarach.v@gmail.com (A.L.); pvegaceledon@gmail.com (P.V.-C.); 2 Millennium Nucleus Bioproducts, Genomics and Environmental Microbiology (BioGEM), Avenida España 1680, Valparaíso 2390123, Chile; michael.seeger@usm.cl; 3 Molecular Microbiology and Environmental Biotechnology Laboratory, Department of Chemistry & Center of Biotechnology Daniel Alkalay Lowitt, Universidad Técnica Federico Santa María, Avda. España 1680, Valparaíso 2390123, Chile

**Keywords:** Botryosphaeria dieback, grapevine trunk diseases, bacterial biocontrol, tissue age, climate variability, seasonality, *Neofusicoccum parvum*, *Diplodia seriata*

## Abstract

Grapevine trunk diseases, particularly Botryosphaeria dieback, pose a major threat to vineyard sustainability, a risk that is further intensified by climate variability and increasing environmental stress. This study evaluated pathogenicity and bacterial biocontrol efficacy against *Neofusicoccum parvum* and *Diplodia seriata* under vineyard conditions, analyzing the combined effects of cultivar (*Vitis vinifera* L. cv. Cabernet Sauvignon and Sauvignon Blanc), tissue type (young shoots and lignified arms), and phenological season (autumn/winter and spring/summer). Pathogenicity assays revealed clear tissue-age specialization: *N. parvum* was more aggressive in young shoots, whereas *D. seriata* caused the most severe vascular lesions in lignified wood. Seasonality further modulated disease expression, with higher lesion development during spring/summer, particularly for *N. parvum* in young shoots, while *D. seriata* maintained high aggressiveness in lignified tissues across both seasons. Berry assays provided a rapid initial assessment of isolate virulence but did not fully reflect pathogen behavior in woody tissue under field conditions. Biological treatments using native bacterial strains (*Pseudomonas* sp. AMCR2b, GcR15a, and *Rhodococcus* sp. PU4) significantly reduced lesion severity in *V. vinifera* under field conditions, although efficacy varied by tissue type and season. Biocontrol effects were generally more stable in lignified arms, and under high disease pressure, only the most robust strains maintained consistent protection, in some cases matching or surpassing the efficacy of the fungicide tebuconazole. These results show that both pathogenicity and biocontrol performance against Botryosphaeria dieback in *V. vinifera* under field conditions are strongly influenced by tissue type and season, supporting bacterial biocontrol as a sustainable component of integrated disease management in vineyards.

## 1. Introduction

*Vitis vinifera* is among the most vital perennial crops globally, crucial for both wine production and the fresh fruit market. However, the longevity and sustainability of vineyards are increasingly threatened by grapevine trunk diseases (GTDs), which can reduce yields by up to 50% and lead to early vine decline or replacement [1,2,3]. Among these diseases, dieback caused by *Botryosphaeriaceae*—particularly *Neofusicoccum parvum* and *Diplodia seriata*—stands out as a widespread and significant problem impacting both young and mature vineyards under Mediterranean climate conditions [2,4,5,6]. The development and severity of these diseases are strongly influenced by environmental stresses such as water deficits and temperature extremes [7], as well as by pruning wounds, which serve as primary entry points for pathogens into the vascular tissues [7,8,9,10,11,12,13].

Cabernet Sauvignon and Sauvignon Blanc are among the most widely cultivated grapevine cultivars worldwide and are key to the production of premium wines in many viticultural regions. Both cultivars are extensively planted in Mediterranean and semi-arid climates, where GTDs are highly prevalent, and their susceptibility to *Botryosphaeriaceae* has been documented under vineyard conditions [1,6,13,14]. Nevertheless, comparative field studies evaluating these cultivars under identical environmental conditions, while simultaneously considering tissue age and seasonal variation, remain limited.

The high pathogenicity of *N. parvum* and *D. seriata* has been widely reported; however, their relative aggressiveness depends strongly on the experimental system and the age of the infected tissue. Early pathogenicity studies, largely based on detached shoots or young plants, consistently identified *N. parvum* as one of the most aggressive *Botryosphaeriaceae* species [5,8,15]. In contrast, more recent field-based studies have demonstrated that *D. seriata* can exhibit equal or greater aggressiveness in mature, lignified wood, particularly in older arms and trunks [13]. These findings highlight the limitations of extrapolating pathogenicity rankings from young tissues to perennial vineyard systems and emphasize the importance of evaluating host–pathogen interactions directly under field conditions. While cultivar susceptibility is not always evident under controlled assays, vineyard evaluations have revealed variability among cultivars, reinforcing the need to study these interactions across contrasting seasons and tissues [15,16,17,18,19]. Therefore, evaluating pathogen aggressiveness across tissues of contrasting age under field conditions is essential to accurately assess disease severity and management efficacy in vineyards.

At present, the management of GTDs is primarily limited to preventive measures, since no curative treatments are available once pathogens become established in the xylem or cortex tissues. In addition, the withdrawal or strict regulation of broad-spectrum fungicides, such as sodium arsenate and benzimidazole, owing to environmental and human health concerns, has considerably reduced the range of chemical control options [20]. Although demethylation inhibitor (DMI) fungicides, including tebuconazole, are still used for pruning wound protection, their field efficacy is variable and strongly influenced by environmental conditions and pathogen pressure [21,22].

Under this scenario, increasing attention has been directed toward more sustainable disease management strategies. Integrated pest management (IPM) programs have promoted the use of biological control agents (BCAs) as preventive alternatives, which are increasingly recognized for their potential to reduce pathogen pressure while minimizing environmental impact [20,23,24,25,26,27,28]. Field studies indicate that applying beneficial microorganisms can reduce both the incidence and severity of GTDs, although efficacy varies depending on tissue type, pathogen species, cultivar, and environmental conditions [13,21,29,30].

In addition to microbial biocontrol agents, plant-derived bioactive compounds have also been widely investigated for their antimicrobial and regulatory properties, highlighting the broad potential of natural products in disease management [31]. In particular, bacteria belonging to the phylum Actinomycetota and species of the genus *Pseudomonas* have repeatedly shown strong antagonistic activity against *Botryosphaeriaceae* and other GTD-associated fungi under laboratory, greenhouse, and field conditions [18,19,32,33,34,35].

Despite growing interest in microbial biocontrol, the combined effects of grapevine cultivar, tissue type, and phenological season on both pathogen aggressiveness and biocontrol performance remain poorly understood under vineyard conditions. In particular, field studies comparing pathogenicity and biological control across cultivars with contrasting susceptibility and across tissues of different ages within the same experimental framework are scarce. Therefore, this study aims to assess the pathogenicity of *N. parvum* and *D. seriata* on young shoots and lignified arms, and to evaluate the preventive efficacy of native bacterial strains and consortia in *V. vinifera* cv. Cabernet Sauvignon and Sauvignon Blanc under vineyard conditions. By explicitly integrating tissue type (young shoots versus lignified arms) and phenological season (autumn/winter versus spring/summer), this work provides field-based evidence on how pathogen behavior and biocontrol effectiveness are shaped by context-dependent and climate-related factors, supporting the role of bacterial biocontrol as a sustainable component of integrated management strategies for Botryosphaeria dieback.

## 2. Results

### 2.1. Pathogenicity in Grapevine Berries

**Figure 1:**
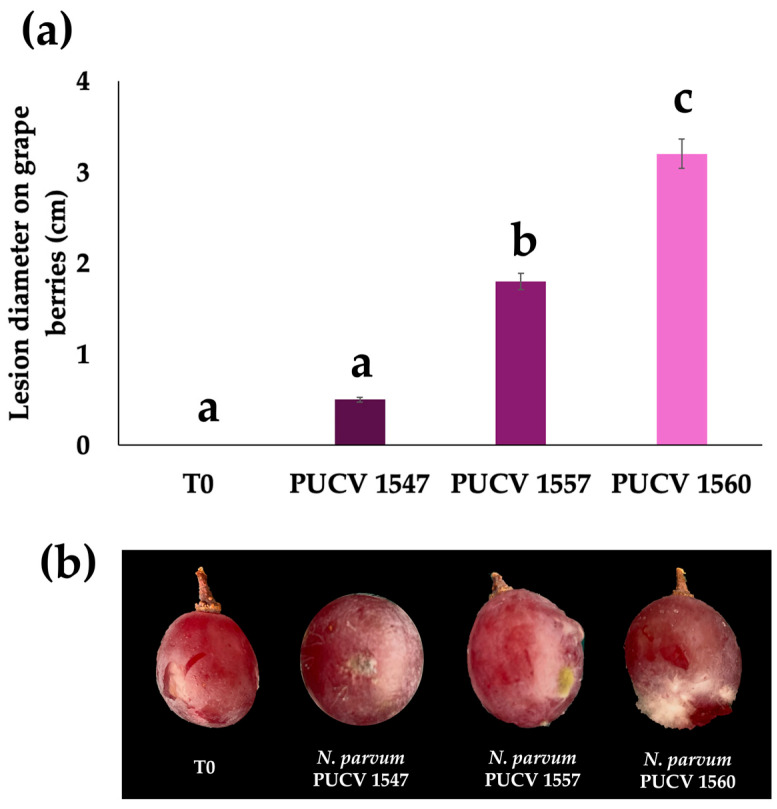
Pathogenicity of *N. parvum* isolates in grape berries (*V. vinifera*). (**a**) Lesion diameter (cm) produced by *N. parvum.* (**b**) Representative images of inoculated berries. Images were taken, and lesions were evaluated 15 days after inoculation. T0 corresponds to wounded, non-inoculated berries. Different letters indicate significant differences according to Tukey’s test (*p* ≤ 0.05), and bars represent the mean ± SD (*n* = 10).

The pathogenicity of *N. parvum* isolates was evaluated by inoculation trials on *V. vinifera* berries, with lesion diameter (cm) recorded after 15 days of incubation in a humid chamber (Figure 1).

In the case of *N. parvum*, Figure 1a,b show significant differences in lesion diameter among the isolates evaluated. Isolate PUCV 1560 exhibited the greatest aggressiveness, reaching values close to 3.2 cm, followed by PUCV 1557, while PUCV 1547 showed significantly less lesion development. The control treatment (T0, uninoculated berries) did not show visible symptoms of rot. Representative images (b) show greater fungal colonization and tissue necrosis in the more aggressive isolates.

### 2.2. Pathogenicity in Grapevines

**Figure 2:**
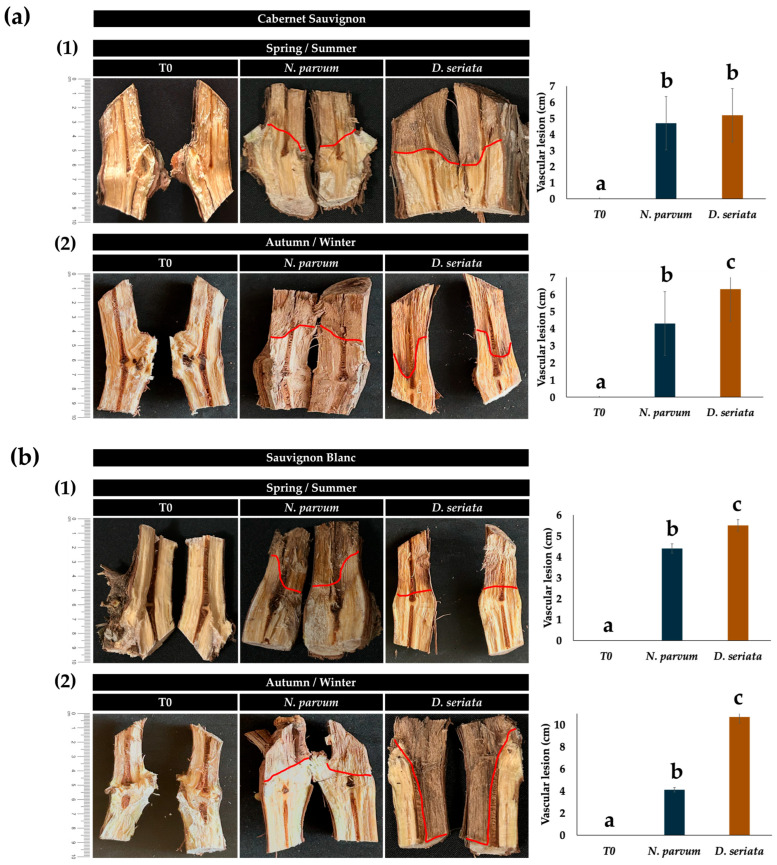
Pathogenicity of *N. parvum* and *D. seriata* on grapevine arms under field conditions. (**a**) Lesion development (cm) on lignified arms of *V. vinifera* cv. Cabernet Sauvignon (**1**) Spring/summer season (**2**) Autumn/winter season. (**b**) Lesion development (cm) on lignified arms of *V. vinifera* cv. Sauvignon Blanc (**1**) Spring/summer season (**2**) Autumn/winter season. Red lines indicate lesion length. T0 corresponds to injured but uninoculated tissue. Bars represent the mean ± SD (*n* = 5). Different letters indicate statistically significant differences between treatments within the same variety, tissue type, and season, as determined by Tukey’s test (*p* ≤ 0.05).

**Figure 3:**
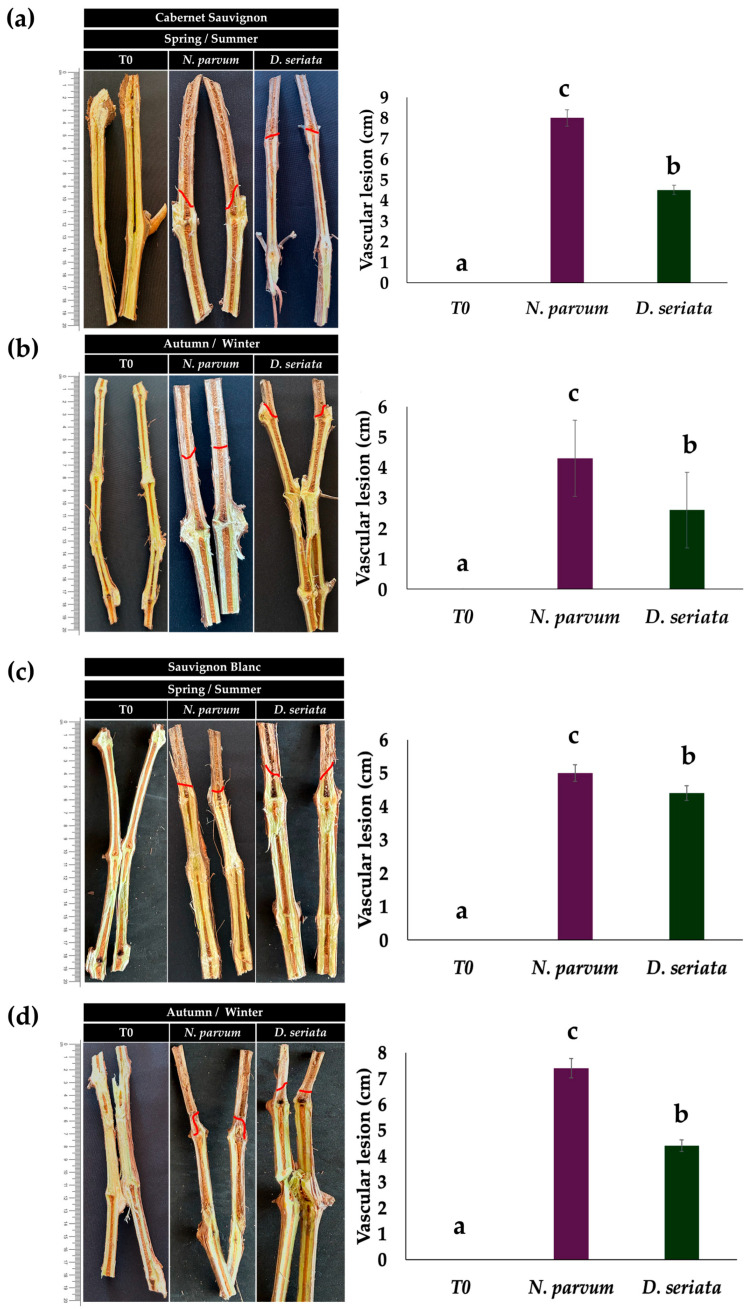
Pathogenicity of *N. parvum* and *D. seriata* on grapevine shoots under field conditions. (**a**) Lesion development (cm) on shoots of *V. vinifera* cv. Cabernet Sauvignon during the spring/summer season. (**b**) Lesion development (cm) on shoots of *V. vinifera* cv. Cabernet Sauvignon during the autumn/winter season. (**c**) Lesion development (cm) on shoots of *V. vinifera* cv. Sauvignon Blanc during the spring/summer season. (**d**) Lesion development (cm) on shoots of *V. vinifera* cv. Sauvignon Blanc during the autumn/winter season. The photographs shown next to each graph correspond to representative shots of the same treatments used for the quantitative measurements. The bar graphs represent the mean lesion length (cm) measured on individual shoots (*n* = 5). T0 corresponds to lesioned but uninoculated tissue. The bars represent the mean ± SD. Different letters indicate statistically significant differences between treatments within the same variety, tissue type, and season, according to Tukey’s test (*p* ≤ 0.05).

Pathogenicity assays revealed clear differences in aggressiveness between the two fungal species, depending on the tissue type evaluated. *N. parvum* caused more extensive lesions in young tissues, whereas *D. seriata* showed greater severity in mature wood. In shoots, *N. parvum* induced lesions with average lengths of approximately 8 cm in Cabernet Sauvignon and 4 cm in Sauvignon Blanc, while *D. seriata* produced shorter lesions, averaging 4.8 cm and 4 cm, respectively. In contrast, in lignified arms, *D. seriata* caused more severe vascular damage, particularly in Cabernet Sauvignon, where lesion lengths reached up to 10.7 cm, compared with an average of 4 cm for *N. parvum*. These results suggest differential tissue preferences: *N. parvum* shows greater aggressiveness in young tissue, while *D. seriata* shows a greater affinity for mature wood (Figure 2 and Figure 3). 

### 2.3. Biocontrol in Grapevines

Field trials showed significant differences in the severity of Botryosphaeria dieback symptoms among biological, chemical, and control treatments (Table 1 and Table 2, Figures S1 and S2). Overall, the bacterial strains evaluated showed a consistent ability to reduce the development of vascular lesions caused by *N. parvum* and *D. seriata*, in both young shoots and lignified arms of *V. vinifera*.

#### 2.3.1. Cabernet Sauvignon

In Cabernet Sauvignon, biological treatments showed significant differences in reducing the severity of vascular lesions caused by *N. parvum* and *D. seriata*, both in young shoots and lignified arms (Table 1, Figures S3 and S5).

Biocontrol of *N. parvum* during the autumn/winter season, the application of the bacterial consortium (GcR15a + AMCR2b) and the endophytic strain PU4 resulted in a marked reduction in lesion length in 2-year-old shoots when compared with the pathogen-inoculated control. Lesions in these treatments ranged from approximately 1.1 to 2.9 cm, whereas the negative control reached approximately 4.3 cm (Table 1 and Figure S3). As expected, the lowest lesion values were observed in the chemical control treated with tebuconazole, where lesion lengths remained below 1 cm. In 8-year-old arms, the biological treatments also limited lesion development, with mean values ranging between approximately 2.5 and 3.6 cm, which were significantly lower than those recorded for the negative control. These results indicate that the biological treatments retained their effectiveness in more lignified tissues during this season.

In spring/summer, *N. parvum* showed an overall increase in disease severity across all treatments (Table 1). Nevertheless, both the consortium and PU4 continued to provide partial protection on shoots, with lesion lengths ranging from approximately 3 to 4.6 cm, and remained significantly shorter than those in the negative control, which reached approximately 8 cm. In lignified arms, the efficacy of the biological treatments was slightly reduced, comparable to that in the autumn/winter period; however, lesion lengths remained limited to approximately 2.0–3.4 cm, values comparable to those obtained with the chemical control and significantly lower than those observed in the pathogen-inoculated control. During the autumn/winter season, biological treatments applied to shoots resulted in a moderate but significant reduction in lesion length compared with the negative control (Table 1). Lesions in treated shoots ranged from approximately 2.8 to 4.5 cm, whereas the negative control reached mean values of around 4.8 cm. In lignified arms, both the bacterial consortium and the PU4 strain effectively reduced lesion development, with lesion lengths ranging from approximately 2 to 4.9 cm, maintaining significant differences relative to the control, which showed lesion lengths close to 5.9 cm (Figure S5).

In spring/summer, *D. seriata* showed a stronger effect of the biological treatments on shoots (Table 1). The consortium achieved its highest efficacy during this period, reducing lesion length to approximately 0.94 cm, while PU4 reduced lesion length to approximately 0.58 cm, both significantly lower than that recorded for the negative control (approximately 2.6 cm). In arms, PU4 also contributed to a reduction in lesion severity, with lesion lengths ranging between approximately 2.2 and 4.0 cm. These values were comparable to those obtained with the fungicide treatment and remained significantly lower than the lesions observed in the pathogen-inoculated control.

#### 2.3.2. Sauvignon Blanc

In Sauvignon Blanc, lesion severity was consistently higher than that observed in Cabernet Sauvignon, suggesting a higher susceptibility of this cultivar to *Botryosphaeriaceae* infections. Nevertheless, the application of biological treatments resulted in measurable reductions in disease in both seasons evaluated (Table 2, Figures S4 and S6). 

For biocontrol of *N. parvum* during the autumn/winter period, both the consortium and the PU4 strain markedly reduced lesion development in shoots when compared with the pathogen-inoculated control. Lesion lengths in treated shoots ranged from approximately 1.1 to 1.8 cm, whereas the negative control reached values close to 7.4 cm (Table 2). In lignified arms, these treatments also limited disease progression, with lesion lengths of approximately 2.5 and 3.6 cm, which were significantly lower than those observed in the negative control (approximately 5.1 cm), indicating effective protection in mature tissue. In spring/summer, disease control in shoots was less consistent. In particular, the consortium treatment did not result in a clear reduction in lesion length, with values close to 6.8 cm. In contrast, PU4 maintained a significant protective effect, reducing lesion length to approximately 2.7 cm compared with the negative control, which averaged around 5 cm. In arms, both biological treatments continued to limit lesion development, maintaining values below 3.4 cm and remaining significantly lower than those recorded for the negative control (approximately 4.4 cm). These results indicate that biocontrol efficacy was more stable in lignified tissues (Figure S6).

For biocontrol of *D. seriata* during autumn/winter, both PU4 and the consortium reduced shoot lesion length relative to the negative control (Table 2). Lesion values in treated shoots ranged from approximately 2.6 to 4.0 cm, whereas the control averaged around 4.6 cm. In lignified arms, the effect of the biological treatments was more pronounced, with lesion lengths close to 3 cm, compared with values exceeding 5.7 cm in the pathogen-inoculated control. In spring/summer, both treatments maintained shoot lesion lengths near 2 cm, which remained significantly lower than those observed in the negative control (approximately 4.4 cm). In arms, PU4 emerged as the most effective treatment, reducing lesion length to approximately 2.7 cm. This performance exceeded that of the consortium and was comparable to the chemical control (Table 2 and Figure S4).

## 3. Discussion

The berry grape pathogenicity assay (Figure 1) confirmed the isolates’ virulence and enabled rapid detection of differences in aggressiveness. However, vineyard results indicate that these patterns do not necessarily reflect pathogen behavior in perennial plants. Previous studies have reported relatively low disease severity in artificially inoculated berries, at around 20%, suggesting a limited capacity of mature fruits to be colonized when compared with other tissues [36]. This contrasts with fruit-specialized pathogens such as *Botrytis cinerea*, which exhibit higher colonization efficiency in mature berries [36]. 

In the case of *D. seriata* PUCV 2021, PUCV 2142, and PUCV 2183, its behavior as a slow colonizer of fruit tissues has already been described and discussed [13], where limited growth and reduced lesion progression in berries were observed, comparable to that seen for *N. parvum*. In both pathogens, the low aggressiveness in fruit contrasts with the severity expressed in woody tissues, reinforcing the idea that the fruit does not constitute an optimal substrate for the full expression of their pathogenicity.

*D. seriata* has been described as a relatively slow colonizer of fruit tissues, and the production of phytotoxic secondary metabolites by this species appears to depend on specific substrate conditions, which may contribute to the lower variability observed among isolates when evaluated in fruit tissues rather than in woody organs [37]. Importantly, previous studies have shown that differences in pathogenicity observed in fruits do not always mirror those in shoots or arms [18,19]. Another important aspect is the use of conidial mixture suspensions in field trials to determine pathogenic behavior representative of field conditions.

**Figure 4:**
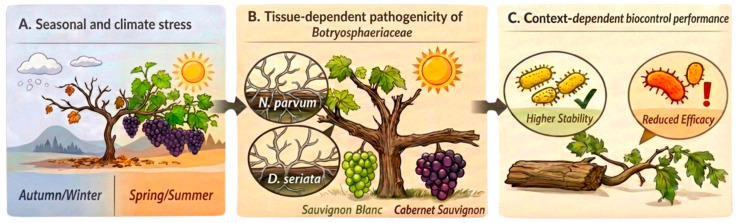
Conceptual model illustrating the interaction between climate conditions, pathogen aggressiveness, and biocontrol performance in Botryosphaeria dieback of grapevine. (**A**) Seasonal climatic dynamics, contrasting autumn/winter and spring/summer conditions. (**B**) Tissue-dependent pathogenicity of *Botryosphaeriaceae*, with *N. parvum* in young shoots and *D. seriata* causing more severe damage in lignified wood, with cultivar-dependent differences. (**C**) Context-dependent performance of bacterial biocontrol, showing higher stability in lignified tissues and reduced efficacy in young shoots under high epidemiological pressure. Created in BioRender. BioGEM L2 (2026) https://BioRender.com/2ko515p (accessed on 28 January 2026).

Field pathogenicity assays revealed a clear specialization of the type of pathogen according to tissue age, highlighting a high-risk scenario for mature wood. In particular, *N. parvum* showed greater aggressiveness in young shoots, whereas *D. seriata* caused the most severe vascular lesions in lignified arms, especially in Cabernet Sauvignon, where lesions reached 10.7 ± 0.4 cm (Figure 2 and Figure 3, Table 2, Figures S3 and S5). This pattern is consistent with the concept that *Botryosphaeriaceae* aggressiveness depends on the degree of tissue maturity and lignification provided by [13], who showed that *D. seriata* expresses markedly higher virulence in older, lignified wood than in young tissues, challenging earlier classifications based primarily on assays conducted on detached canes or young plants. While early studies frequently identified *N. parvum* as the most aggressive *Botryosphaeriaceae* species (this tissue-specific pathogenic behavior is schematically summarized in Figure 4B) [4,5], these assessments were primarily based on young plant material, highlighting the significance of tissue age in interpreting pathogenicity under vineyard conditions.

The discrepancies observed relative to other studies probably reflect differences in vineyard conditions, including water stress, vine vigor, wound exposure, and microclimate, as well as the timing of inoculation in relation to seasonal host activity. Fernández et al. [7] demonstrated that short but intense heat events combined with water scarcity markedly increase lesion development caused by *Botryosphaeriaceae*, enhancing pathogen colonization and symptom expression under field conditions. This is especially relevant here, as the highest disease severity and lowest biocontrol effectiveness occurred during spring-summer, associated with periods of high maximum temperatures recorded at the EELP vineyard [38]. These results support the idea that thermal stress significantly exacerbates Botryosphaeria dieback in Mediterranean climates [3,7,11,15,39]. 

These pathogenicity patterns may help explain differences in biological control performance. In this study, Sauvignon Blanc tended to show greater disease severity than Cabernet Sauvignon. This was evident, for example, in the negative control for *N. parvum* in shoots during autumn/winter, where lesions reached 7.4 cm in Sauvignon Blanc, compared with approximately 4.3 cm in Cabernet Sauvignon, indicating a markedly higher susceptibility. This pattern is consistent with field observations reported by Úrbez-Torres et al. [1] and Pitt et al. [6], who documented cultivar-dependent differences in *Botryosphaeriaceae* symptom expression under vineyard conditions. It also agrees with Castillo-Novales et al. [19], who showed that *N. parvum* progresses more rapidly in young tissues and that biocontrol efficacy depends strongly on the biological context and bacterial strain.

The greater susceptibility seen in Sauvignon Blanc may stem from specific physiological and structural traits unique to the cultivar. Variations among *V. vinifera* cultivars in aspects such as wood anatomy, lignification levels, wound compartmentalization ability, and profiles of phenolics and stilbenes—either naturally present or induced—are believed to play crucial roles in how GTD manifests [17,40,41]. Cultivars that are less capable of quickly sealing xylem vessels or producing antimicrobial secondary metabolites might enable pathogens to spread more rapidly through vascular tissues. Additionally, host responses to environmental stresses such as heat and water scarcity can interact with these innate defenses, potentially worsening disease outcomes in more vulnerable cultivars, especially under Mediterranean conditions [7,21]. Although this study did not directly measure these physiological traits, the consistent patterns observed across various tissues and seasons support the idea that original cultivar traits influence susceptibility to Botryosphaeria dieback.

The spring/summer season functioned as a high epidemiological pressure scenario, under which some biological effects weakened. This was particularly evident in Sauvignon Blanc shoots infected with *N. parvum*, where the bacterial consortium reached lesion lengths of 6.8 cm (0% inhibition), whereas strain PU4 maintained a significant protective effect (2.7 cm; 46% inhibition). Rather than representing inconsistency, this pattern should be interpreted as biologically meaningful and is conceptually illustrated in Figure 4C under conditions favorable for pathogen development, treatment differences narrow and only the most robust colonizers or competitors maintain their performance [21,29,42,43].

Most vineyard-scale field studies on grapevine trunk diseases have focused on preventive protection of pruning wounds and on evaluating treatments across different vineyards and seasons rather than through simultaneous comparisons among cultivars and tissue types within a single experimental framework [11,12,29]. Although some studies have addressed cultivar susceptibility or tissue age effects, these factors are rarely evaluated together under controlled field conditions [1,13]. In this context, the explicit comparison between Cabernet Sauvignon and Sauvignon Blanc, as well as between young shoots and lignified arms across two phenological seasons, with two different *Botryosphaeriaceae* species, addresses an important knowledge gap by assessing how cultivar susceptibility and tissue maturity jointly influence pathogenicity and biocontrol performance under real vineyard conditions. From an applied perspective, these results indicate that a single management strategy-either one biocontrol agent or one application timing-is unlikely to be optimal across cultivars and infection windows [21,39].

In both cultivars, biological treatments reduced vascular lesions caused by *N. parvum* and *D. seriata*; however, their efficacy depended strongly on tissue type and phenological season (Table 1 and Table 2). This context dependency aligns with patterns widely reported in vineyard field studies, in which control levels vary across plant compartments, pathogen pressure, and environmental conditions during infection and treatment [11,12,39]. 

Vineyard-scale evaluations focused on pruning wound protection provide an additional framework for interpreting these results. In a European multisite study, fungicides achieved mean disease control levels ranging from 44% to 95% against *D. seriata*, whereas Trichoderma-based biological products did not significantly reduce infection compared with inoculated controls [29]. These findings confirm that, although biological tools can be effective in specific contexts, consistency under field pressure remains a major challenge in perennial systems such as vineyards [11,12,44]. 

In the present study, biological treatments were generally more stable in lignified arms than in young shoots, particularly in Sauvignon Blanc. In arms infected with *N. parvum*, *Rhodococcus* sp. PU4 consistently reduced lesion length across both seasons, remaining below the level of the negative control. This higher stability may be explained by improved bacterial persistence in lignified tissues, lower tissue turnover, and a more buffered microenvironment compared with actively growing shoots [45]. Such persistence may reflect better niche adaptation and greater competitive ability within mature woody tissues. Regulatory mechanisms linked to biofilm formation, cyclic di-GMP metabolism, and extracellular polymeric substance production have been shown to modulate bacterial colonization efficiency in *Pseudomonas* spp. [46], suggesting that similar regulatory pathways may contribute to the stability observed in lignified arms under vineyard conditions [47,48,49,50,51]. 

This pattern is consistent with studies demonstrating that biocontrol efficacy depends strongly on tissue type, temperature, and pathogen genotype. Recent assays have shown wide variability in *D. seriata* inhibition across different experimental conditions and thermal ranges, with isolate-specific responses [18,19]. Collectively, these results reinforce the view that biocontrol efficacy is not an intrinsic trait of a given agent, but rather the outcome of complex interactions among host, environment, and pathogen.

The bacterial strains *Pseudomonas* sp. AMCR2b and GcR15a and *Rhodococcus* sp. PU4 significantly reduced lesions in *V. vinifera* under field conditions caused by *N. parvum* and *D. seriata*. The control results were comparable to the fungicide tebuconazole, particularly in summer shoot assays. The performance of the bacterial consortium suggests a potential synergistic effect among strains with complementary modes of action [52]. Regulatory genes associated with biofilm dynamics and c-di-GMP signaling have been shown to coordinate siderophore production, motility, and exopolysaccharide synthesis in *Pseudomonas* spp. [46], traits that may enhance competitive colonization of pruning wounds. The strong performance of strain PU4 expands the range of microorganisms considered as biocontrol agents in viticulture [18,45,53,54]. 

The interactions among climatic conditions, pathogen aggressiveness, and biocontrol performance observed in this study under field conditions are summarized in the conceptual framework presented in Figure 4. Seasonal thermal dynamics, particularly short but intense heat events, act as key drivers of host stress and pathogen behavior in vineyards. Pathogen aggressiveness becomes strongly dependent on tissue type and cultivar, with *N. parvum* predominance in young shoots and *D. seriata* causing more severe damage in lignified wood. At the same time, increasing thermal stress reduces the reliability of bacterial biocontrol, whereas only the most robust antagonists maintain protective effects during periods of high epidemiological pressure. This framework highlights climate as a central factor linking disease development and biocontrol performance under vineyard conditions.

Overall, building on the conceptual framework outlined in Figure 4, these results validate the potential of native antagonistic bacteria as sustainable tools for managing *Botryosphaeria* dieback under real vineyard conditions. Nevertheless, an effective application method of these biocontrol agents should be determined. The use of these bacteria in integrated disease management programs could help reduce fungicide inputs, preserve native microbiota, and increase the resilience of viticultural systems to climate change. Future research should focus on linking field performance to the underlying molecular and metabolomic mechanisms, including induced plant defenses and bioactive compound production.

## 4. Materials and Methods

### 4.1. Chemicals Reagents, and Culture Media 

D-Glucose was obtained from Merck (Darmstadt, Germany). Yeast extract and Bacto proteose peptone No. 3 were purchased from Difco Laboratories (Franklin Lakes, NJ, USA). Malt extract and potato dextrose agar (PDA) were obtained from HiMedia Laboratories (West Chester, PA, USA).

### 4.2. Microorganisms

The bacterial *Pseudomonas* strains utilized in this study were previously isolated by Vega-Celedón et al. [55], and the Actinomycete *Rhodococcus* was isolated by Larach et al. [2] (Table 3). The fungal isolates *N. parvum* and *D. seriata* were obtained by Larach et al. [2] (Table 4). The selection process and preliminary evaluation of these microorganisms as biocontrol agents are described in Larach et al. [18] and Castillo-Novales et al. [19].

### 4.3. Pathogenicity Tests on Berry Grapes

The inoculations were performed on fresh Red Globe grapes of *V. vinifera*. The grapes were purchased from the market in the summer of 2024 and used immediately for the test. Berries were disinfected with 0.1% sodium hypochlorite for 30 s and rinsed three times with sterile distilled water (SDW). A 5 mm diameter mycelial plug from a 6-day-old PDA culture was inoculated into the center of each berry through a wound created with a sterile needle. Pathogenicity assays were conducted using three fungal isolates of *N. parvum* (PUCV 1547, PUCV 1557, PUCV 1560). The control treatment (T0) consisted of wounded but uninoculated berries (*n* = 10). Berries were incubated individually for 15 days in humid chambers at room temperature. Following incubation, the extent of fruit rot was measured at the inoculation site.

### 4.4. Field Trial

#### 4.4.1. Inoculum Preparation

Fungal spore suspensions were prepared. A mix of *N. parvum* PUCV 1547, PUCV 1557, and PUCV 1560, and a mix of *D. seriata* PUCV 2120, PUCV 2142, and PUCV 2183 were according to the method described by Marsh et al. [56], modified by Castillo-Novales et al. [19]. A 5-day-old mycelium agar disc was placed on plates containing 2% water-agar and autoclaved pine needles. The plates were incubated under near-UV light (λ = 320 nm) until pycnidia and conidia formed. Mature pycnidia were macerated in sterile distilled water, and the solution was filtered through sterile gauze. A conidial concentration was determined using a hemocytometer and adjusted to 1 × 10^4^ conidia µL^−1^.

The bacterial solution was prepared according to the method described by Coman et al. [57], modified by Vega-Celedón et al. [55]. The bacteria were cultured in Yeast Malt (YM; 10 g L^−1^ glucose, 3 g L^−1^ malt extract, 5 g L^−1^ peptone, 3 g L^−1^ yeast extract) for 24 h at room temperature, collected, and resuspended in Falcon tubes with 30 mL of YM. The absorbance was adjusted to 0.2 at 600 nm to obtain a final concentration of 1 × 10^8^ CFU mL^−1^.

#### 4.4.2. Field Trial

The experiment was conducted on Cabernet Sauvignon and Sauvignon Blanc vines in an experimental vineyard at the La Palma Experimental Station of the PUCV. The selected vineyard showed no prior symptoms of GTDs and was managed using a bilateral cordon training system, with spur pruning to four buds. All vines used in the trial were non-grafted plants. Inoculations were performed on 2-year-old shoots, and the cut was made at the four-bud level. Immediately, the shoots were inoculated with 50 µL of each bacterial suspension in YM medium, YM medium alone as a negative control (C−), and 50 µL of tebuconazole was applied as a commercial formulation containing 23% (*w*/*v*) active ingredient, diluted to a final concentration of 0.5% (*w*/*v*), as a positive control (C+). After 24 h, the shoots were inoculated with 50 µL of the fungal spore suspension at the same end where the previous inoculation had been performed. The arms were inoculated with a mixture of *N. parvum* spores and another mixture of *D. seriata* spores and subjected to their respective treatments (consortium of GcR15a and AMCR2b strains, endophytic strain PU4), the negative control (C−), or the chemical control (C+) (*n* = 5).

#### 4.4.3. Assessment and Recovery of Pathogen-Induced Damage

The length of vascular lesions was assessed during the 2023 autumn/winter and 2024 spring/summer seasons. Damage to each tissue was evaluated in the field, and plant material was cut to measure the lesions (the assay was performed in duplicate). To recover the fungus, tissue samples were taken from the lesion progression area, disinfected with 1% sodium hypochlorite for 5 s, washed three times with deionized water (SDW), and cultured on APDA medium to complete Koch’s postulates. *N. parvum* and *D. seriata* were identified morphologically by examining the shape of the conidia under a microscope, following the methodology described by Larach et al. [2]. The inhibition percentage of each treatment was calculated using Equation (1) [58].

(1) $$\mathrm{Percentage}\;\mathrm{of}\;\mathrm{inhibition}\;(\%)=\left(\frac{\left(R-r\right)}{R}\right)*100$$

where *R* represents the mean lesion length (cm) observed in the inoculated control (pathogen only), and *r* represents the mean lesion length (cm) recorded in treated shoots or arms (biocontrol or chemical treatments) [58].

### 4.5. Statistical Analysis

Data were analyzed using a one-way analysis of variance (ANOVA) [59] to study the effects of the biological control strains. When the assumptions of homogeneity of variance (Shapiro–Wilk test) were not met, a nonparametric analysis of variance using the Kruskal–Wallis test was performed. Data means were compared using Tukey’s test to detect significant differences (*p* ≤ 0.05) [60] using Infostat software version 2017. The field trials were completely randomized, with five replicates per treatment.

### 4.6. Conceptual Figure Development (BioRender)

Figure 4 was developed to conceptualize and integrate the main findings of this study, illustrating the interactions among climatic conditions, pathogen aggressiveness, tissue type, and biocontrol performance in grapevine Botryosphaeria dieback. The figure was created using BioRender, where individual graphical elements were selected and assembled to represent the experimental framework and biological processes addressed in this research.

To ensure visual consistency and clarity, the final composition was refined using the Apply BioRender Style tool, which harmonizes graphical elements within the platform. BioRender was used exclusively as a visualization tool to support the interpretation and communication of experimental results. BioRender license: https://BioRender.com/2ko515p (accessed on 28 January 2026).

## 5. Conclusions

This study demonstrates that pathogenicity and biocontrol efficacy against Botryosphaeria dieback under vineyard conditions are strongly influenced by cultivar, tissue maturity, and phenological season. *N. parvum* was more aggressive in young shoots, whereas *D. seriata* caused more severe lesions in lignified wood, confirming a clear tissue age specialization associated with host structure.

Biological treatments significantly reduced vascular lesion development caused by both pathogens, although their effectiveness varied by tissue type and season. Biocontrol effects were generally more stable in lignified arms, and only the most robust strains maintained consistent protection under high epidemiological pressure. Native bacterial *Pseudomonas* sp. strains AMCR2b and GcR15a and *Rhodococcus* sp. PU4 showed strong potential as a biocontrol agent under field conditions.

Overall, these findings support the need for integrated and context-specific disease management strategies that consider cultivar susceptibility, tissue type, and infection period, and reinforce the role of native bacterial biocontrol as a sustainable tool for managing grapevine trunk diseases in vineyards.

## Figures and Tables

**Table 1:** Effects of biological and chemical treatments on lesion severity caused by *Botryosphaeriaceae* in *V. vinifera* cv. Cabernet Sauvignon during two phenological seasons (lesion length in cm; Inhibition percentage %).

Treatments		Autumn/Winter		Spring/Summer	
		cm	Inhibition (%)	cm	Inhibition (%)
2-year-old shoots	T0	0 ^c^	-	0 ^d^	-
	*N. parvum*	4.3 ± 0.4 ^a^	0	8 ± 3.5 ^a^	0
	*N. parvum* + Consortium	1.1 ± 0.5 ^c^	74	3.7 ± 1.2 ^c^	54
	*N. parvum* + Tebuconazole	3.9 ± 0.3 ^a^	9	5.1 ± 2.1 ^b^	36
	*N. parvum* + PU4	2.9 ± 0.6 ^b^	33	4.6 ± 1.2 ^b^	43
	T0	0 ^d^	-	0 ^c^	-
	*D. seriata*	2.6 ± 1.1 ^a^	0	4.5 ± 3.6 ^a^	0
	*D. seriata* + Consortium	0.9 ± 0.1 ^c^	65	2.8 ± 1.8 ^b^	38
	*D. seriata* + Tebuconazole	0.3 ± 0.2 ^a^	0	6 ± 1.9 b^c^	0
	*D. seriata* + PU4	0.6 ± 0.4 ^b^	77	3 ± 1.4 ^b^	33
8-year-old arms	T0	0 ^d^	-	0 ^d^	-
	*N. parvum*	4.3 ± 0.2 ^a^	0	4.7 ± 0.8 ^a^	0
	*N. parvum* + Consortium	3.6 ± 0.2 ^b^	16	2.7 ± 1.1 ^c^	43
	*N. parvum* + Tebuconazole	2.4 ± 0.3 ^c^	44	2.1 ± 1.4 ^c^	55
	*N. parvum* + PU4	4.1 ± 0.3 ^a^	5	3.4 ± 0.7 ^b^	28
	T0	0 ^c^	-	0 ^c^	-
	*D. seriata*	6.3 ± 0.4 ^a^	0	5.2 ± 0.8 ^a^	0
	*D. seriata* + Consortium	4.9 ± 0.3 ^a^	17	2.2 ± 0.4 ^b^	58
	*D. seriata* + Tebuconazole	0.9 ± 0.7 ^c^	85	2.6 ± 0.5 ^b^	50
	*D. seriata* + PU4	2.8 ± 0.7 ^b^	53	4 ± 2.1 ^a^	23

Data are expressed as mean ± standard deviation (*n* = 5); superscript letters indicate statistically significant differences compared to the control (Tukey’s test, *p* ≤ 0.05).

**Table 2:** Effects of biological and chemical treatments on lesion severity caused by *Botryosphaeriaceae* in *V. vinifera* cv. Sauvignon Blanc during two phenological seasons (lesion length in cm; Inhibition percentage %).

Treatments		Autumn/Winter		Spring/Summer	
		cm	Inhibition (%)	cm	Inhibition (%)
2-year-old shoots	T0	0 ^c^	-	0 ^d^	-
	*N. parvum*	7.4 ± 0.5 ^a^	0	5 ± 1.5 ^a^	0
	*N. parvum* + Consortium	1.1 ± 0.6 ^bc^	85	6.8 ± 2.5 ^a^	0
	*N. parvum* + Tebuconazole	0.6 ± 0.4 ^c^	92	3.6 ± 1.3 ^b^	28
	*N. parvum* + PU4	1.8 ± 0.3 ^b^	76	2.7 ± 0.8 ^c^	46
	T0	0 ^d^	-	0 ^d^	-
	*D. seriata*	4.4 ± 0.7 ^a^	0	4.4 ± 1.9 ^a^	0
	*D. seriata* + Consortium	2.1 ± 0.3 ^b^	52	2.6 ± 1.3 ^b^	41
	*D. seriata* + Tebuconazole	1.4 ± 0.7 ^c^	68	4 ± 1.9 ^c^	9
	*D. seriata* + PU4	1.4 ± 0.4 ^c^	68	1.4 ± 1.9 ^c^	68
8-year-old arms	T0	0 ^d^	-	0 ^d^	-
	*N. parvum*	4.1 ± 0.4 ^a^	0	4.4 ± 1.7 ^a^	0
	*N. parvum* + Consortium	3.6 ± 0.4 ^b^	29	3.4 ± 1.5 ^b^	23
	*N. parvum* + Tebuconazole	0.5 ± 0.5 ^d^	91	2.6 ± 0.9 ^c^	41
	*N. parvum* + PU4	2.5 ± 0.3 ^c^	51	2.8 ± 0.9 ^c^	36
	T0	0 ^d^	-	0 ^d^	-
	*D. seriata*	10.7 ± 0.4 ^a^	0	5.5 ± 1.9 ^a^	0
	*D. seriata* + Consortium	3.5 ± 0.4 ^b^	39	3.5 ± 2.5 ^b^	36
	*D. seriata* + Tebuconazole	0.8 ± 0.5 ^d^	87	1.3 ± 0.7 ^c^	76
	*D. seriata* + PU4	2.9 ± 0.3 ^c^	49	2.7 ± 0.7 ^b^	51

Data are expressed as mean ± standard deviation (*n* = 5); superscript letters indicate statistically significant differences compared to the control (Tukey’s test, *p* ≤ 0.05).

**Table 3:** Biocontrol bacterial strains.

Biocontroller	First Match of 16S rRNA Partial Gene Sequence	Identity (%)	Access No. GenBank
*Pseudomonas* sp. AMCR2b	*Pseudomonas asgharzadehiana* strain SWRI132	689/689 (100%)	OQ244037
*Pseudomonas* sp. GcR15a	*Pseudomonas orientalis* strain R4-35-08	706/707 (99.8%)	MW548343
*Rhodococcus* sp. PU4	*Rhodococcus* sp. strain H-cryo-48	682/685 (99.5%)	OQ244039

**Table 4:** *Botryosphaeriaceae* fungal isolates.

Isolate		Locality (Commune, Region, Chile)	Access No. GenBank	
			ITS	BT
*N. parvum*	PUCV 1547	Peralillo, O’Higgins	KM870224	KP762483
	PUCV 1557	Palmilla, O’Higgins	KM870225	KP762484
	PUCV 1560	Talca, Maule	KM870226	KP762485
*D. seriata*	PUCV 2120	Palmilla, O’Higgins	MT023573	MT063140
	PUCV 2142	Batuco, Maule	MT023574	MT063141
	PUCV 2183	Pencahue, Maule	MT023587	MT063154

## Data Availability

The original contributions presented in the study are included in the article; further inquiries can be directed to the corresponding authors.

## Supplementary Materials

The following supporting information can be downloaded at: https://www.mdpi.com/article/10.3390/plants15050728/s1, Figure S1: Field trials, pathogenicity, and biocontrol trials were conducted during the autumn/winter season in a *V. vinifera* vineyard. (A-B) General view of a row trial in *V. vinifera* cv. Sauvignon Blanc vineyard. (C,D) General view of a row trial in a *V. vinifera* cv. Cabernet Sauvignon vineyard. (E,F) Marking and identification of inoculated shoots and arms with the different treatments in the vineyard in March 2023; Figure S2: Experimental pathogenicity and biocontrol trial conducted during the spring/summer season in a *V. vinifera* vineyard. (A) General view of the vineyard with different varieties during the spring/summer period, characterized by higher temperatures and active vegetative growth. (B) Vines emerging from dormancy into spring. (C,D) Rows of the vineyard during the evaluation period, highlighting the experimental design and the identification of inoculated shoots and arms; Figure S3: Efficacy of biocontrol in lignified shoots of *V. vinifera* cv. Cabernet Sauvignon under field conditions. (a) Representative longitudinal sections of grapevine shoots inoculated in the spring/summer season with a mixed suspension of *N. parvum* and a mixed suspension of *D. seriata.* (b) Representative longitudinal sections of grapevine shoots inoculated in the autumn/winter season with a mixed suspension of *N. parvum* and a mixed suspension of *D. seriata.* Treatments of the wounded but non-inoculated control (T0), chemical control with tebuconazole (C+), bacterial consortium (*Pseudomonas* sp. AMCR2b + GcR15a), and the endophytic strain *Rhodococcus* sp. PU4, and inoculated controls without treatment (C-). Abbreviations: NP: *Neofusicoccum parvum*; DS: *Diplodia seriata*. Red lines indicate the extent of internal vascular necrosis used to assess disease severity; Figure S4: Efficacy of biocontrol in lignified shoots of *V. vinifera* cv. Sauvignon Blanc under field conditions. (a) Representative longitudinal sections of grapevine shoots inoculated in the spring/summer season with a mixed suspension of *N. parvum* and a mixed suspension of *D. seriata.* (b) Representative longitudinal sections of grapevine shoots inoculated in the autumn/winter season with a mixed suspension of *N. parvum* and a mixed suspension of *D. seriata.* Treatments of the wounded but non-inoculated control (T0), chemical control with tebuconazole (C+), bacterial consortium (*Pseudomonas* sp. AMCR2b + GcR15a), and the endophytic strain *Rhodococcus* sp. PU4, and inoculated controls without treatment (C-). Abbreviations: NP: *Neofusicoccum parvum*; DS: *Diplodia seriata*. Red lines indicate the extent of internal vascular necrosis used to assess disease severity; Figure S5: Efficacy of biocontrol in lignified arms of *V. vinifera* cv. Cabernet Sauvignon under field conditions. (a) Representative longitudinal sections of grapevine arms inoculated in the spring/summer season with a mixed suspension of *N. parvum*. (b) Longitudinal sections of grapevine arms inoculated in the spring/summer season with a mixed suspension of *D. seriata.* (c) Longitudinal sections of grapevine arms inoculated in the autumn/winter season with a mixed suspension of *N. parvum*. (d) Longitudinal sections of grapevine arms inoculated in the autumn/winter season with a mixed suspension of *D. seriata.* Treatments of the wounded but non-inoculated control (T0), chemical control with tebuconazole (C+), bacterial consortium (*Pseudomonas* sp. AMCR2b + GcR15a), and the endophytic strain *Rhodococcus* sp. PU4, and inoculated controls without treatment (C-). Red lines indicate the extent of internal vascular necrosis used to assess disease severity; Figure S6: Efficacy of biocontrol in lignified arms of *V. vinifera* cv. Sauvignon Blanc under field conditions. (a) Representative longitudinal sections of grapevine arms inoculated in the spring/summer season with a mixed suspension of *N. parvum*. (b) Longitudinal sections of grapevine arms inoculated in the spring/summer season with a mixed suspension of *D. seriata.* (c) Longitudinal sections of grapevine arms inoculated in the autumn/winter season with a mixed suspension of *N. parvum*. (d) Longitudinal sections of grapevine arms inoculated in the autumn/winter season with a mixed suspension of *D. seriata.* Treatments of the wounded but non-inoculated control (T0), chemical control with tebuconazole (C+), bacterial consortium (*Pseudomonas* sp. AMCR2b + GcR15a), and the endophytic strain *Rhodococcus* sp. PU4, and inoculated controls without treatment (C-). Red lines indicate the extent of internal vascular necrosis used to assess disease severity.

## Abbreviations

The following abbreviations are used in this manuscript:

GTDs	grapevine trunk diseases
DMI	demethylation inhibitor
IPM	integrated pest management
BCAs	biological control agents
cm	centimeters
PUCV	Pontificie Universidad Católica de Valparaíso
SD	standard deviation
EELP	Estación Experimental La Palma
ITS	internal transcribed spacer region
BT	β-tubulin gene
SDW	sterile distilled water
nm	nanometers
µL	microliter
*w*/*v*	weight/volume
PDA	potato dextrose agar
APDA	acidified potato dextrose agar
