# Supplementary material for: Seasonal Effects on Pathogenicity and Biocontrol Management of Botryosphaeria Dieback in Vitis vinifera L. cv. Cabernet Sauvignon and Sauvignon Blanc Under Field Conditions
Source: Plants (Basel). 2026 Feb 27;15(5):728. doi: 10.3390/plants15050728 (PMC12986627; doi:10.3390/plants15050728)
Supplement: Supplementary file 1 [file plants-15-00728-s001.zip › plants-4142615-supplementary.pdf]

## Supplementary material

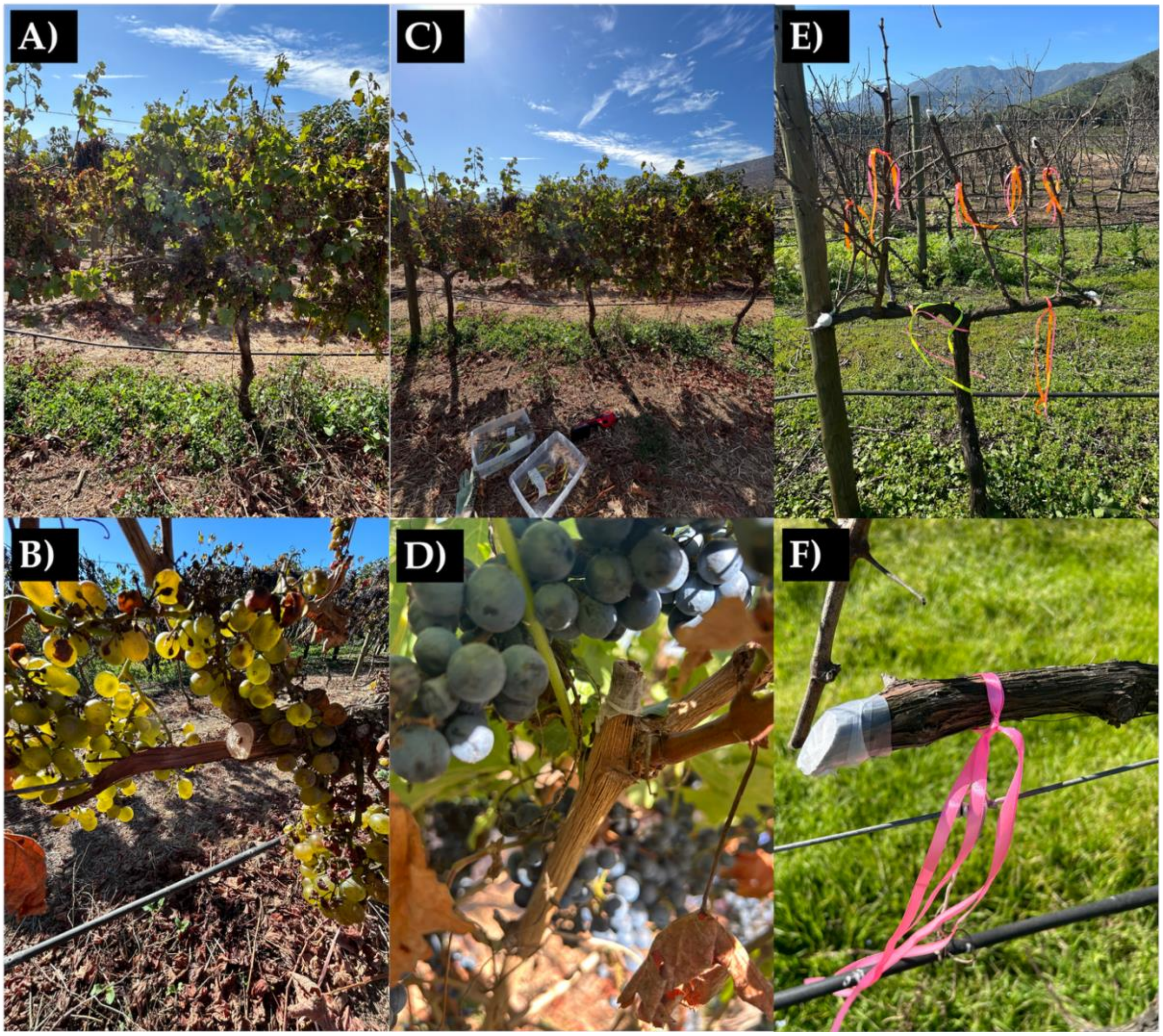


**Figure S1. Field trials, pathogenicity, and biocontrol trials were conducted during the autumn/winter season in a *V. vinifera* vineyard.** (A-B) General view of a row trial in *V. vinifera* cv. Sauvignon Blanc vineyard. (C-D) General view of a row trial in a *V. vinifera* cv. Cabernet Sauvignon vineyard. (E-F) Marking and identification of inoculated shoots and arms with the different treatments in the vineyard in March 2023.

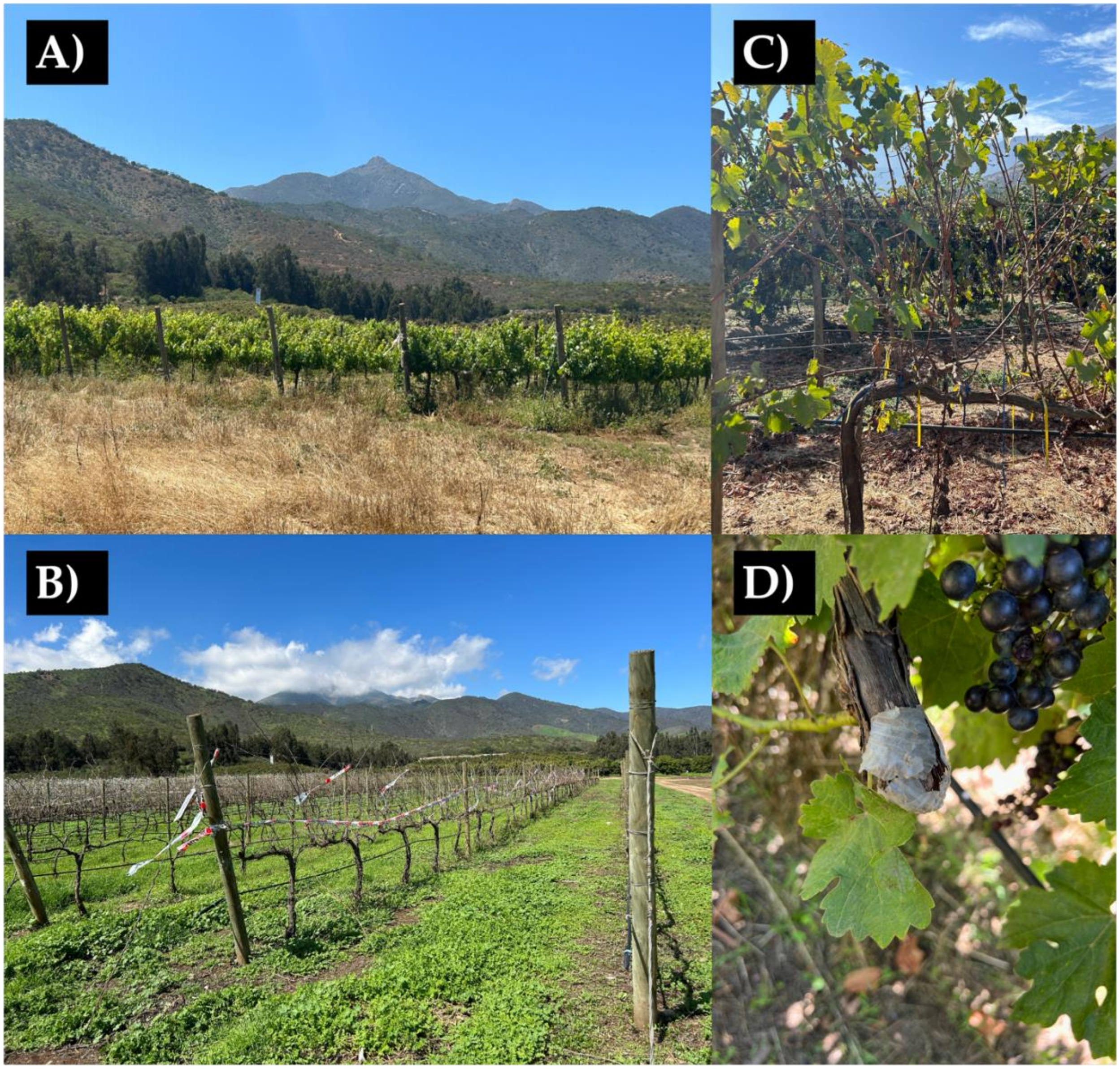


**Figure S2. Experimental pathogenicity and biocontrol trial conducted during the spring/summer season in a *V. vinifera* vineyard.** (A) General view of the vineyard with different varieties during the spring/summer period, characterized by higher temperatures and active vegetative growth. (B) Vines emerging from dormancy into spring. (C-D) Rows of the vineyard during the evaluation period, highlighting the experimental design and the identification of inoculated shoots and arms.

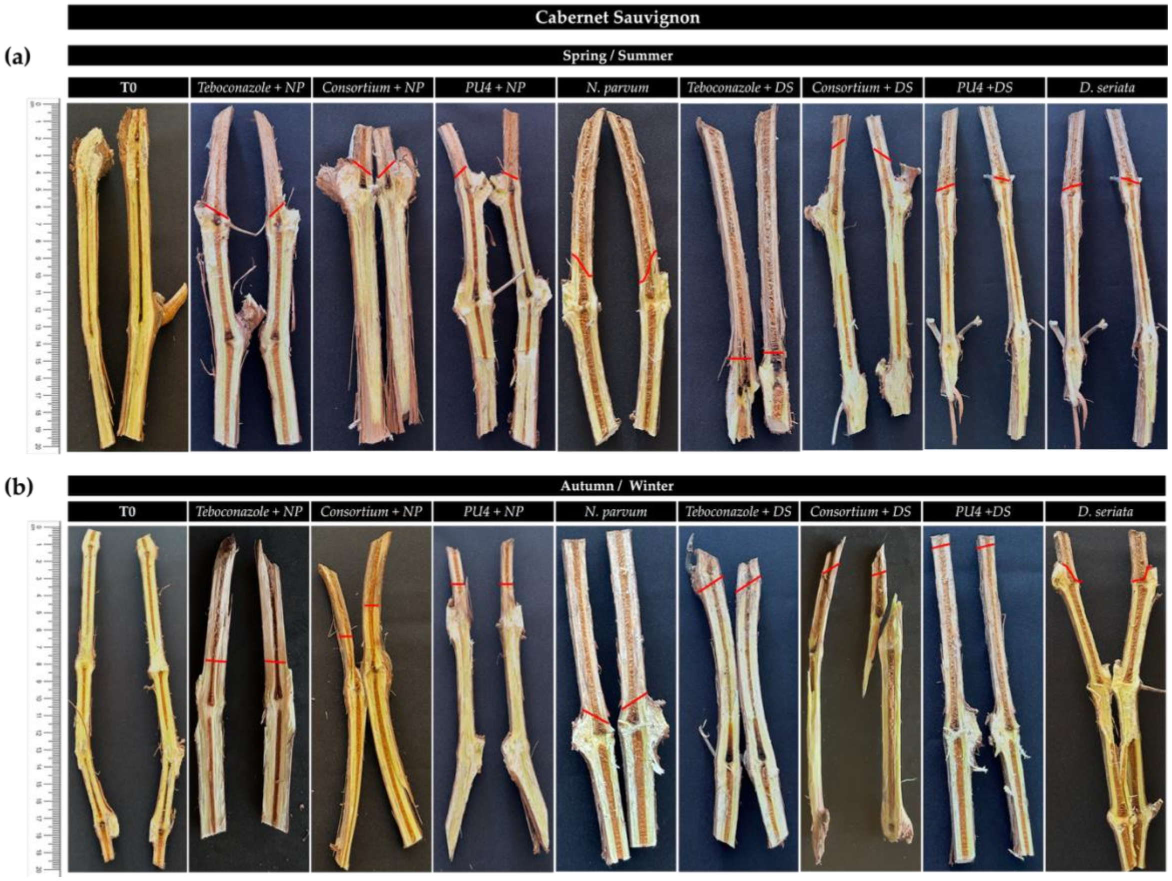


**Figure S3. Efficacy of biocontrol in lignified shoots of *V. vinifera* cv. Cabernet Sauvignon under field conditions**. (a) Representative longitudinal sections of grapevine shoots inoculated in the spring/summer season with a mixed suspension of *N. parvum* and a mixed suspension of *D. seriata.* (b) Representative longitudinal sections of grapevine shoots inoculated in the autumn/winter season with a mixed suspension of *N. parvum* a mixed suspension of *D. seriata*. Treatments of the wounded but non-inoculated control (T0), chemical control with tebuconazole (C+), bacterial consortium (*Pseudomonas* sp. AMCR2b + GcR15a), and the endophytic strain *Rhodococcus* sp. PU4, and inoculated controls without treatment (C-). Abbreviations: NP: *Neofusicoccum parvum*; DS: *Diplodia seriata*. Red lines indicate the extent of internal vascular necrosis used to assess disease severity.

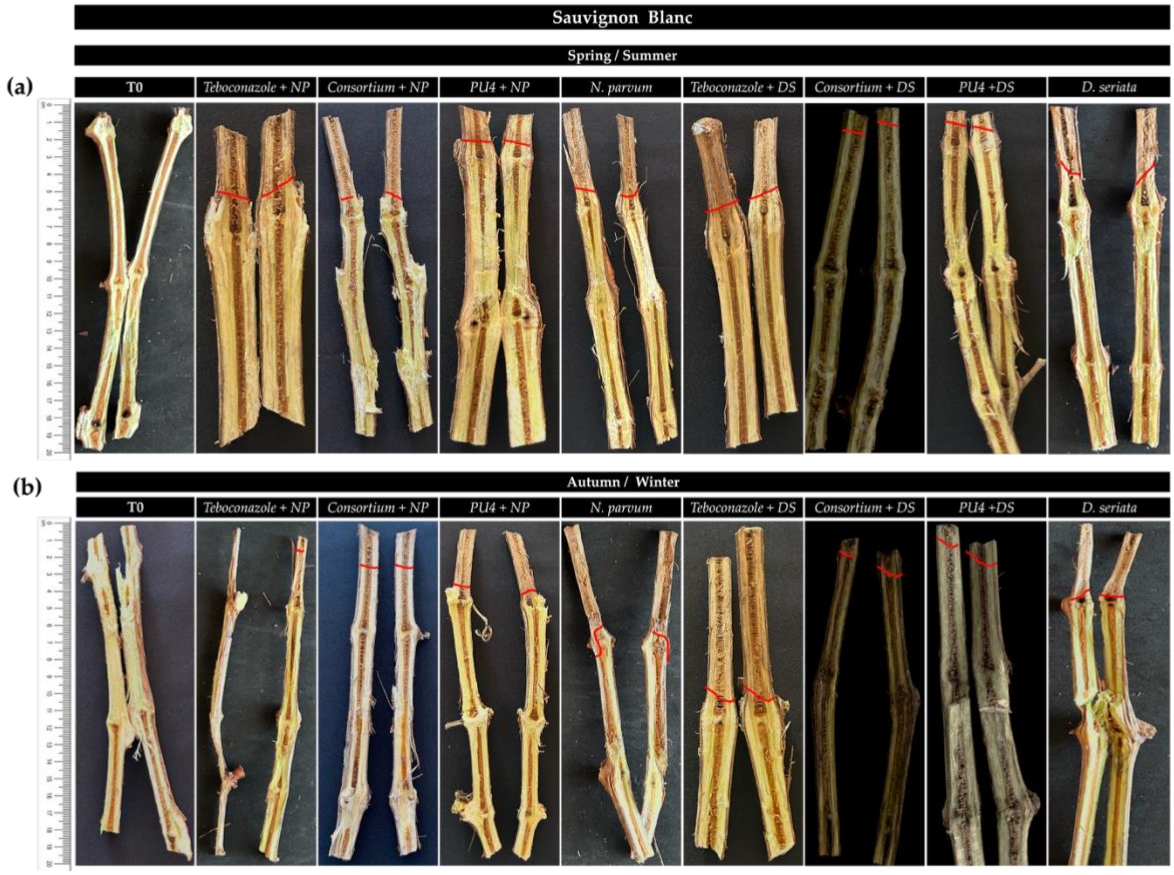


**Figure S4. Efficacy of biocontrol in lignified shoots of *V. vinifera* cv. Sauvignon Blanc under field conditions**. (a) Representative longitudinal sections of grapevine shoots inoculated in the spring/summer season with a mixed suspension of *N. parvum* and a mixed suspension of *D. seriata.* (b) Representative longitudinal sections of grapevine shoots inoculated in the autumn/winter season with a mixed suspension of *N. parvum* a mixed suspension of *D. seriata*. Treatments of the wounded but non-inoculated control (T0), chemical control with tebuconazole (C+), bacterial consortium (*Pseudomonas* sp. AMCR2b + GcR15a), and the endophytic strain *Rhodococcus* sp. PU4, and inoculated controls without treatment (C-). Abbreviations: NP: *Neofusicoccum parvum*; DS: *Diplodia seriata*. Red lines indicate the extent of internal vascular necrosis used to assess disease severity.

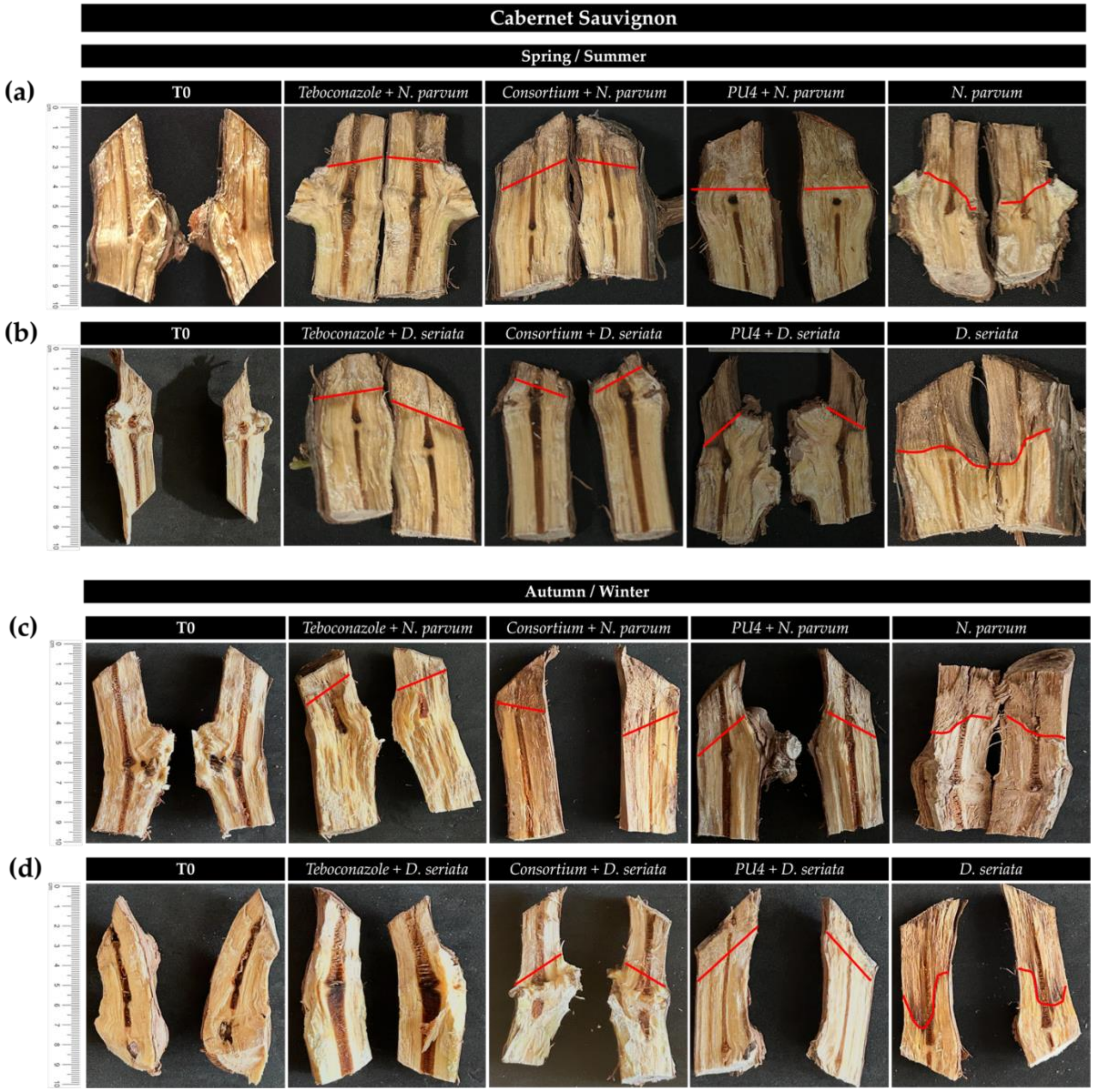


**Figure S5. Efficacy of biocontrol in lignified arms of *V. vinifera* cv. Cabernet Sauvignon under field conditions.** (a) Representative longitudinal sections of grapevine arms inoculated in the spring/summer season with a mixed suspension of *N. parvum*. (b) Longitudinal sections of grapevine arms inoculated in the spring/summer season with a mixed suspension of *D. seriata.* (c) Longitudinal sections of grapevine arms inoculated in the autumn/winter season with a mixed suspension of *N. parvum*. (d) Longitudinal sections of grapevine arms inoculated in the autumn/winter season with a mixed suspension of *D. seriata.* Treatments of the wounded but non-inoculated control (T0), chemical control with tebuconazole (C+), bacterial consortium (*Pseudomonas* sp. AMCR2b + GcR15a), and the endophytic strain *Rhodococcus* sp. PU4, and inoculated controls without treatment (C-). Red lines indicate the extent of internal vascular necrosis used to assess disease severity.

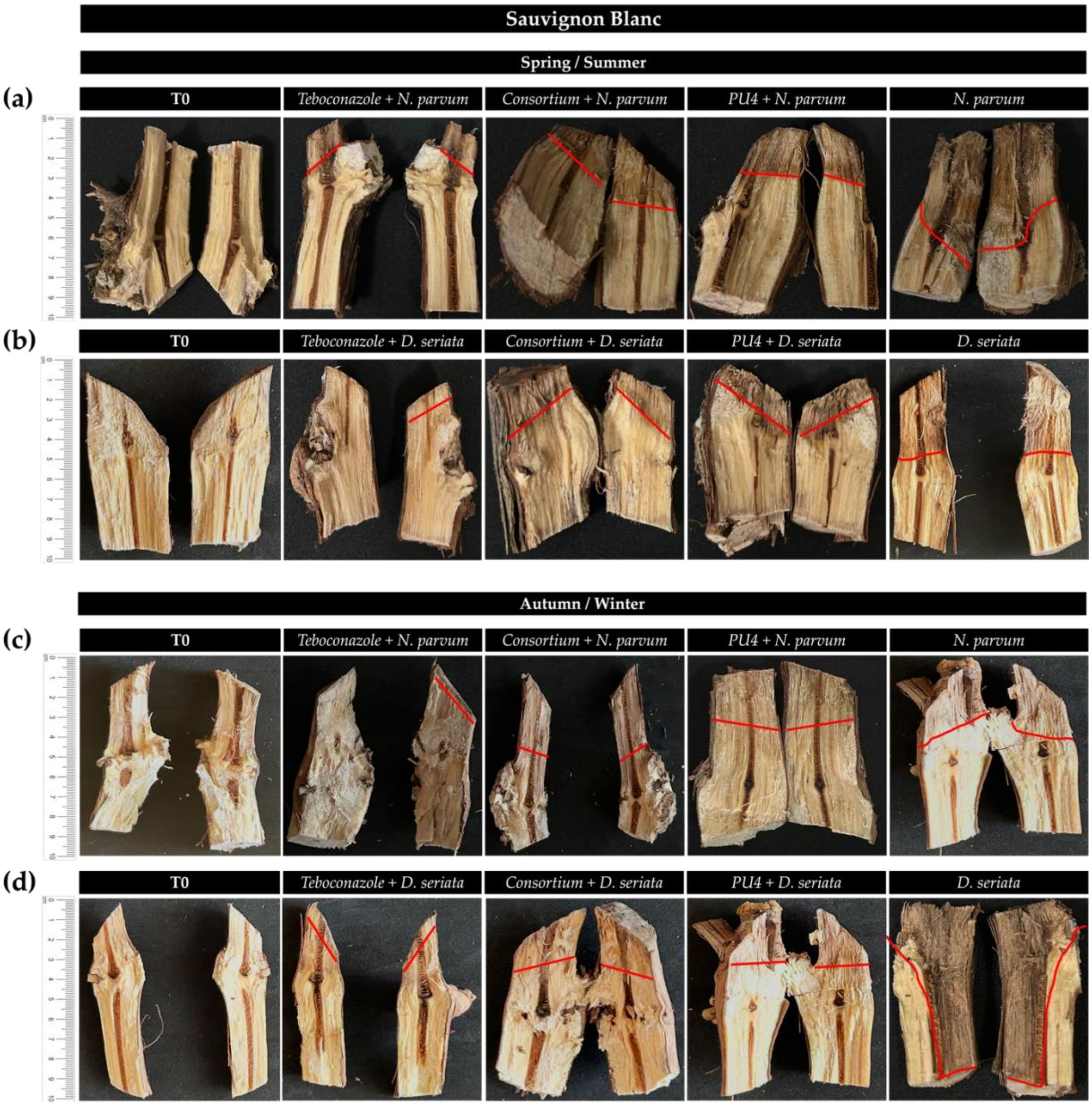


**Figure S6. Efficacy of biocontrol in lignified arms of *V. vinifera* cv. Sauvignon Blanc under field conditions.** (a) Representative longitudinal sections of grapevine arms inoculated in the spring/summer season with a mixed suspension of *N. parvum*. (b) Longitudinal sections of grapevine arms inoculated in the spring/summer season with a mixed suspension of *D. seriata.* (c) Longitudinal sections of grapevine arms inoculated in the autumn/winter season with a mixed suspension of *N. parvum*. (d) Longitudinal sections of grapevine arms inoculated in the autumn/winter season with a mixed suspension of *D. seriata.* Treatments of the wounded but non-inoculated control (T0), chemical control with tebuconazole (C+), bacterial consortium (*Pseudomonas* sp. AMCR2b + GcR15a), and the endophytic strain *Rhodococcus* sp. PU4, and inoculated controls without treatment (C-). Red lines indicate the extent of internal vascular necrosis used to assess disease severity.
